# Biochemical impact of dexmedetomidine on inflammatory and stress markers in gastric cancer surgery: A meta-analysis

**DOI:** 10.5937/jomb0-60059

**Published:** 2026-01-06

**Authors:** Xuhui Zhang, Wenjun Hu, Jinghua Wang, Rui Qin, Xinlei Wang, Zhenhua Zhang

**Affiliations:** 1 The 305 Hospital of PLA, Department of Anesthesiology, Beijing 100017, China; 2 The 305 Hospital of PLA, Department of Gastroenterology, Beijing 100017, China

**Keywords:** dexmedetomidine, inflammatory markers, stress hormones, gastric cancer, meta-analysis, biochemical modulation, deksmedetomidin, inflamatorni markeri, hormoni stresa, rak želuca, meta-analiza, biohemijska modulacija

## Abstract

**Background:**

Dexmedetomidine, an a2-adrenergic agonist, has been reported to modulate inflammatory responses and neuroendocrine stress in surgical settings. This meta-analysis evaluated its effects on serum biochemical markers of inflammation and stress in patients undergoing gastric cancer surgery.

**Methods:**

Literature was retrieved from PubMed, CNKI, Wanfang, and VIP databases. Studies comparing dexmedetomidine anesthesia with conventional regimens in gastric cancer surgery were included. RevMan 5.2 was used for meta-analysis. Outcome indicators included interleukin-6 (IL-6), tumor necrosis factor-a (TNF-a), cortisol, epinephrine, adrenocorticotropic hormone (ACTH), heart rate (HR), mean arterial pressure (MAP), and adverse events.

**Results:**

Fifteen studies were analyzed. Compared with controls, dexmedetomidine significantly reduced serum levels of IL-6, TNF-a, cortisol, epinephrine, and ACTH (all P &lt; 0.00001). It also decreased HR, MAP visual analog scale (VAS) scores, and incidence of adverse reactions.

**Conclusions:**

Dexmedetomidine anesthesia effectively reduces biochemical markers of inflammation and stress in gastric cancer surgery, suggesting its beneficial role in modulating perioperative biochemical responses.

## Abstract (sr)

**Uvod:**

Prijavljeno je da deksmedetomidin, a2-adrenergički agonist, modulira inflamatorne odgovore i neuroendokrini stres u hirurškim uslovima. Ova meta-analiza j'e procenila njegove efekte na serumske biohemijske markere upale i stresa kod pacijenata koji su podvrgnuti operaciji raka želuca.

**Metode:**

Literatura je preuzeta iz baza podataka PubMed, CNKI, Wanfang i VIP. Uključene su studij'e koj'e upoređuju deksmedetomidinsku anesteziju sa konvencionalnim reži-mima u hirurgiji raka želuca. Za meta-analizu je korišćen RevMan 5.2. Indikatori ishoda su uključivali interleukin-6 (IL-6), faktor nekroze tumora-a (TNF-a), kortizol, epinefrin, adrenokortikotropni hormon (ACTH), srčanu frekvencij'u (HR), srednji arterijski pritisak (MAP) i neželjene događaje.

**Rezultati:**

Analizirano je petnaest studija. U poređenju sa kontrolama, deksmedetomidin je značajno smanjio serumske nivoe IL-6, TNF-a, kortizola, epinefrina i ACTH (sve P &lt; 0,00001). Takođe j'e smanjio HR, MAP rezultate vizuelne analogne skale (VAS) i učestalost neželj'enih reak-cija.

**Zaključak:**

Deksmedetomidinska anestezija efikasno sman-juje biohemijske markere upale i stresa kod operacija raka želuca, što ukazuje na njenu korisnu ulogu u modulacij'i perioperativnih biohemijskih odgovora.

## Introduction

Gastric cancer is a common malignant tumor of the digestive tract in clinical practice. Globally, it ranks among the top five in incidence and among the top four in mortality [1] [2].

Radical surgery is a commonly employed treatment method for early-stage gastric cancer and may be performed multiple times. As a key factor directly influencing surgical outcomes, the choice of anesthesia protocol is of critical importance for postoperative wound healing and patient recovery [3].

Dexmedetomidine, by binding to α2-adrenergic receptors, inhibits sympathetic nervous system excitation. It not only provides effective analgesic and sedative effects but also alleviates surgical stress responses and maintains hemodynamic stability [4].

Currently, the efficacy and safety of dexmedetomidine anesthesia in gastric cancer surgery remain under investigation, and no consensus has been reached. Therefore, this study aims to conduct a meta-analysis to evaluate changes in serum inflammatory factors and stress response indicators in gastric cancer patients undergoing surgery with dexmedetomidine anesthesia, thereby providing evidence to support its broader clinical application.

## Materials and methods

### Literature search

Search keywords included: »dexmedetomidine,« »gastric cancer surgery,« »stress response,« »inflammatory factors,« »adverse reactions,« and their corresponding English terms. Relevant literature was retrieved from both Chinese and English databases, including VIP, CNKI, PubMed, and Wanfang Medical, covering the past five years.

Expert consultation was also used to obtain additional references. In cases where included studies had unclear results or missing data, authors were contacted for clarification or supplementary information.

Articles were screened based on titles and keywords to ensure alignment with the research topic and approval by relevant institutional ethics boards. Studies with obvious operational errors, repeated content, or inconsistent methodologies were excluded after preliminary reading. Selected literature was analyzed using RevMan 5.2 software for meta-analysis.

### Inclusion and exclusion criteria

Inclusion criteria: (1) Studies that used dex-medetomidine alone or in combination as the intervention group and compared it to other anesthetic regimens as the control group, with analysis of outcome differences post-intervention; (2) Randomized selection of subjects, without restrictions on nationality or race; (3) Published between 2021 and 2025; (4) Studies with complete clinical data and adherence to the principle of a single research variable.

Exclusion criteria: (1) Reviews, meta-analyses, case reports, or conference abstracts; (2) Cell or animal-based experimental studies; (3) Studies not relevant to the research focus.

### Outcome indicators

A comparison was made between the observation and control groups after intervention with respect to the following indicators: heart rate (HR), mean arterial pressure (MAP), visual analog scale (VAS) score, interleukin-6 (IL-6), tumor necrosis factor-alpha (TNF-α), cortisol (Cor), epinephrine (E), adrenocorticotropic hormone (ACTH), and the incidence of adverse reactions.

### Quality assessment

Study quality was assessed using the modified Jadad scale. A score of 3 indicated low quality, while 4 indicated high quality.

### Statistical analysis

Data were analyzed using RevMan 5.2 software. Count data were expressed as risk ratios (RR), and continuous variables were presented as standardized mean differences (SMD), both with 95% confidence intervals (CI). Heterogeneity was tested using the Chi^2^ test. A random-effects model was applied if P<0.1 and I^2^ 50%; otherwise, a fixed-effects model was used.

## Results

### Literature search results and characteristics

**Figure 1:**
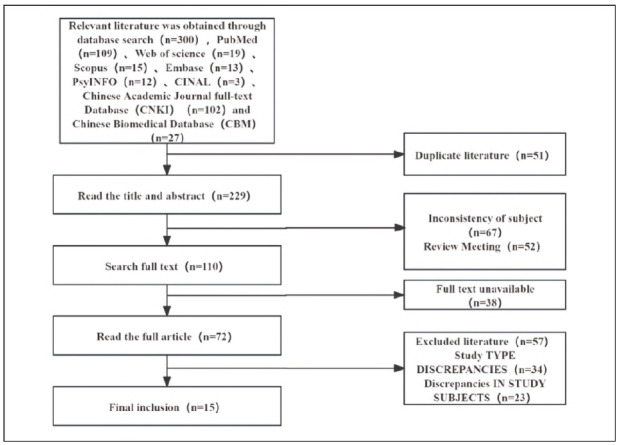
Literature Selection Flowchart.

A total of 280 articles were retrieved from Chinese and English databases including Wanfang, CNKI, and PubMed, based on the study topic and search keywords. After screening according to the inclusion and exclusion criteria, 13 Chinese-language studies and 2 English-language studies were included. The literature selection process is shown in Figure 1.

**Figure 2:**
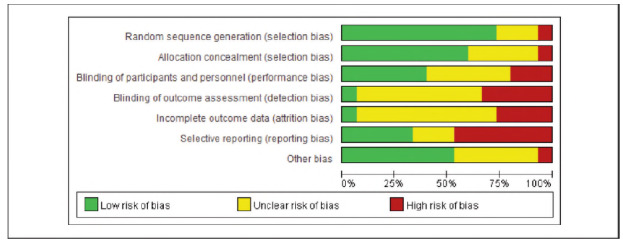
Overall Publication Bias of Included Studies.

**Figure 3:**
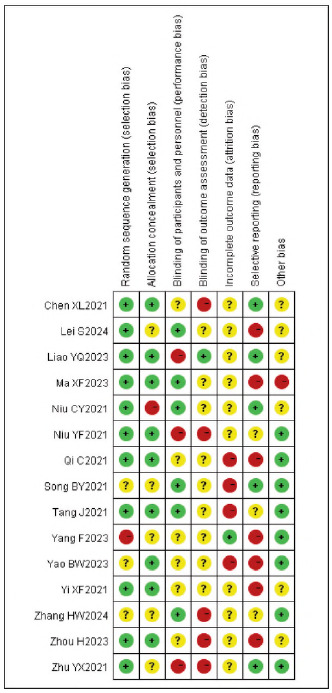
Publication Bias of Individual Studies.

Of the 15 included studies, 12 were rated as high quality and 3 as low quality [5] [6] [7] [8] [6] [9] [10] [11] [12] [13] [14] [15] [16] [17] [18]. No significant publication bias was detected (Figure 2-Figure 3).

### Meta-analysis of hemodynamic indicators with dexmedetomidine

A total of 7 studies reporting MAP and 9 studies reporting HR were included. Heterogeneity analysis showed significant heterogeneity among the included studies for MAP (I^2^ = 84.0%) and HR (I^2^ = 98.0%) (both P < 0.00001). Therefore, a random-effects model was applied. The results showed that the MAP (RR: -7.02, 95% CI: (-8.06, -5.98)) and HR (RR: - 8.07, 95% CI: (-8.71, -7.42)) in the observation group were significantly lower than those in the control group, with all differences being statistically significant (P < 0.00001).

**Figure 4:**
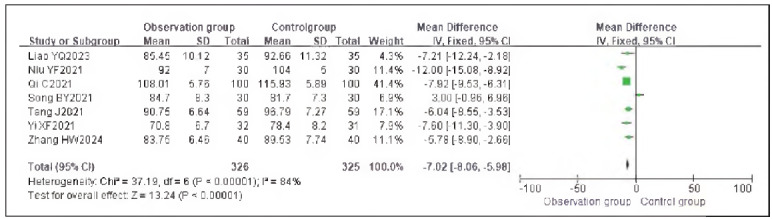
Forest Plot of MAP

**Figure 5:**
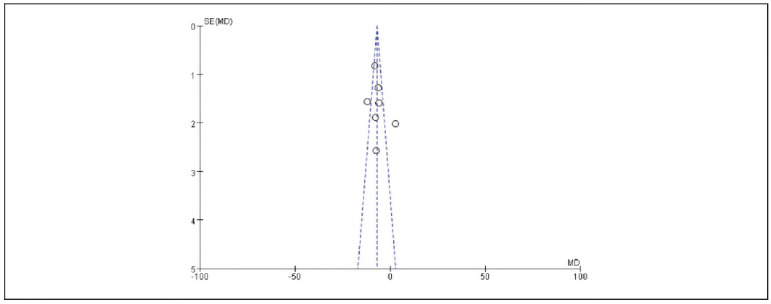
Funnel Plot of MAR

**Figure 6:**
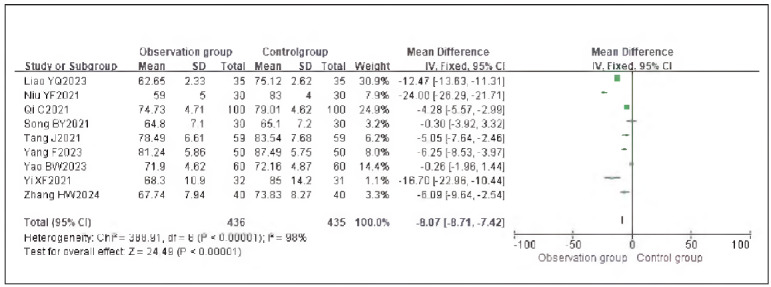
Forest Plot of HR.

**Figure 7:**
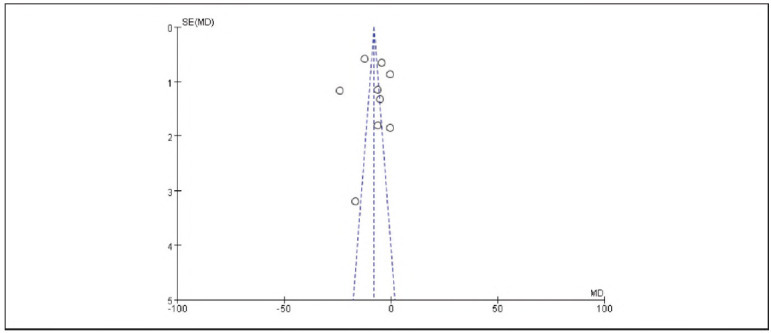
Funnel Plot of HR.

These findings suggest that dexmedetomidine reduces the hemodynamic impact in patients undergoing gastric cancer surgery. See Figure 4, Figure 5, Figure 6-Figure 7.

### Meta-analysis of analgesic indicators with dexmedetomidine

Nine studies reporting VAS scores were included. Heterogeneity analysis showed significant heterogeneity among studies (I^2^ = 95.0%, P < 0.00001). Therefore, a random-effects model was used. The results demonstrated that the VAS scores in the observation group were significantly lower than those in the control group, with a statistically significant difference (RR: -0.66, 95% CI: (-0.70, -0.62), P < 0.00001).

**Figure 8:**
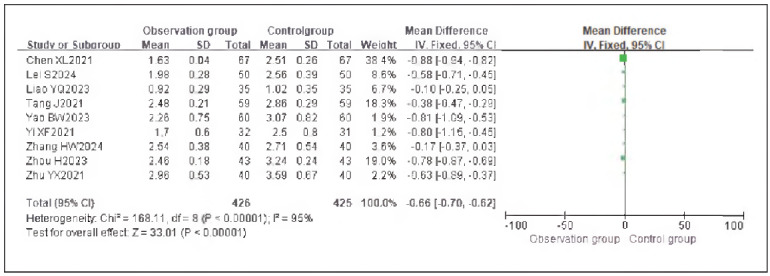
Forest Plot of VAS Scores.

**Figure 9:**
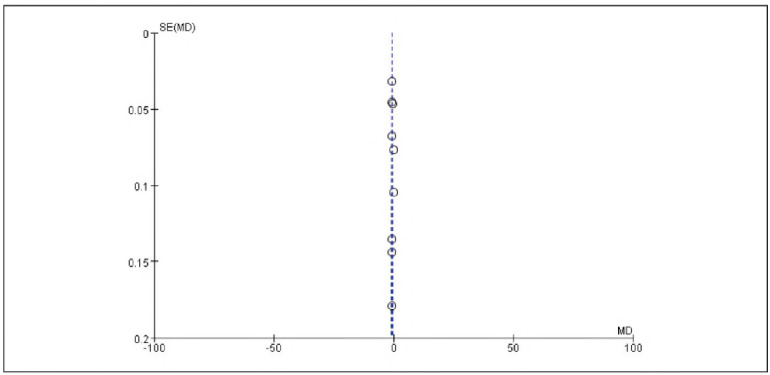
Funnel Plot of VAS Scores.

These findings suggest that dexmedetomidine provides effective analgesia and can reduce pain perception in patients undergoing gastric cancer surgery. See Figure 8-Figure 9.

### Meta-analysis of oxidative stress indicators with dexmedetomidine

Seven studies reporting cortisol (Cor), five studies reporting epinephrine (E), and five studies reporting adrenocorticotropic hormone (ACTH) were included. Heterogeneity analysis showed significant heterogeneity among the studies for Cor (I^2^ = 95.0%), E (I^2^ = 93.0%), and ACTH (I^2^ = 97.0%) (all P < 0.00001).

Using a random-effects model, the results demonstrated that the levels of Cor (RR: -23.55, 95% CI: (-27.19, -19.92)), E (RR: -23.65, 95% CI: (-26.55, -20.64)), and ACTH (RR: -14.34, 95% CI: (-15.66, -13.02)) in the observation group were significantly lower than those in the control group, with statistically significant differences (P < 0.00001).

**Figure 10:**
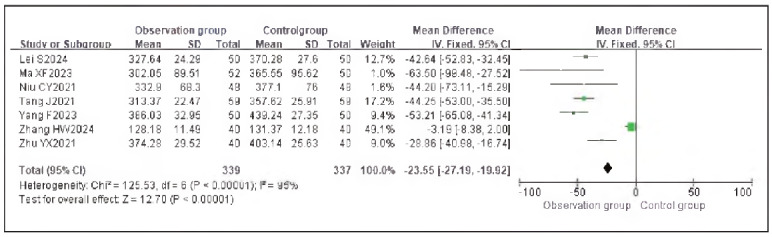
Forest Plot of Cortisol (Cor).

**Figure 11:**
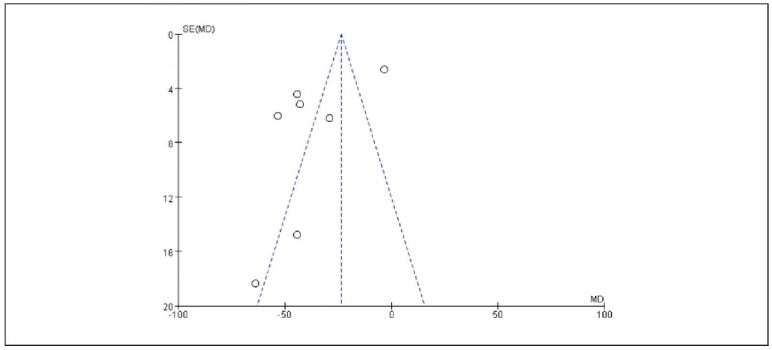
Funnel Plot of Cortisol (Cor).

**Figure 12:**
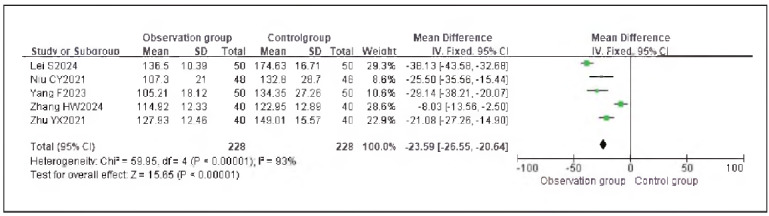
Forest Plot of Epinephrine (E).

**Figure 13:**
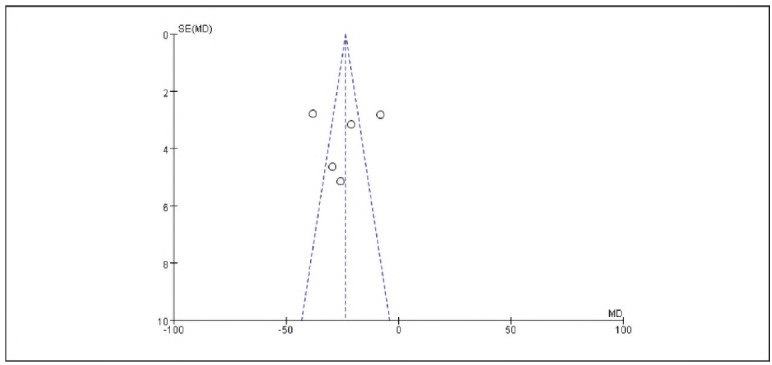
Funnel Plot of Epinephrine (E).

**Figure 14:**
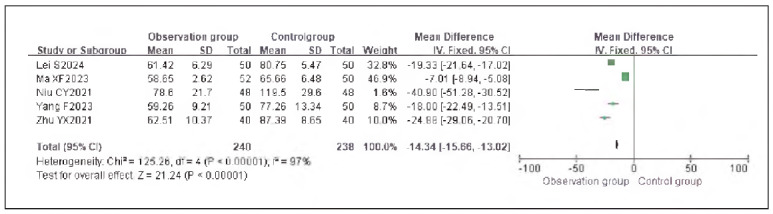
Forest Plot of Adrenocorticotropic Hormone (ACTH).

**Figure 15:**
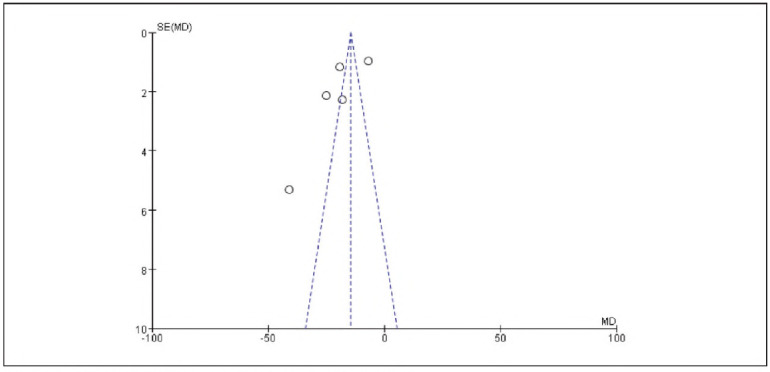
Funnel Plot of Adrenocorticotropic Hormone (ACTH).

These findings suggest that dexmedetomidine alleviates oxidative stress responses in patients undergoing gastric cancer surgery. See Figure 10, Figure 11, Figure 12, Figure 13, Figure 14-Figure 15.

### Meta-analysis of inflammatory indicators with dexmedetomidine

Five studies reporting tumor necrosis factor-alpha (TNF-α) and seven studies reporting interleukin-6 (IL-6) were included. Heterogeneity analysis indicated significant heterogeneity among the studies for TNF-α (I^2^ = 97.0%) and IL-6 (I^2^ = 94.0%) (both P < 0.00001).

Using a random-effects model, the results showed that TNF-α (RR: -7.92, 95% CI: (-8.44, - 7.41)) and IL-6 (RR: -7.66, 95% CI: (-8.21, -7.11)) levels in the observation group were significantly lower than those in the control group, with statistically significant differences (P < 0.00001).

**Figure 16:**
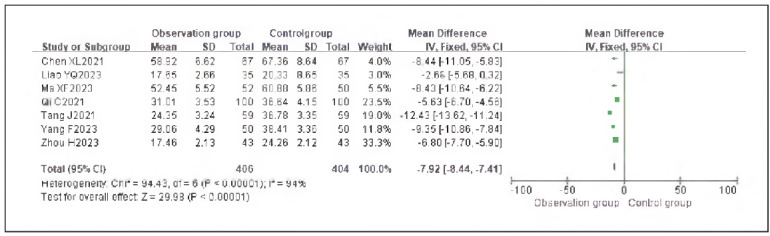
Forest Plot of Tumor Necrosis Factor-alpha (TNF-α).

**Figure 17:**
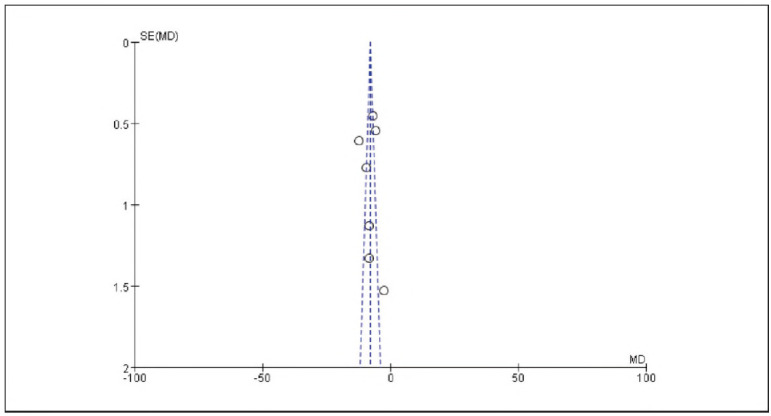
Funnel Plot of Tumor Necrosis Factor-alpha (TNF-α).

**Figure 18:**
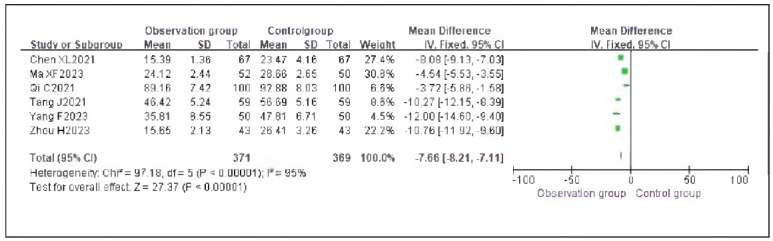
Forest Plot of Interleukin-6 (IL-6).

**Figure 19:**
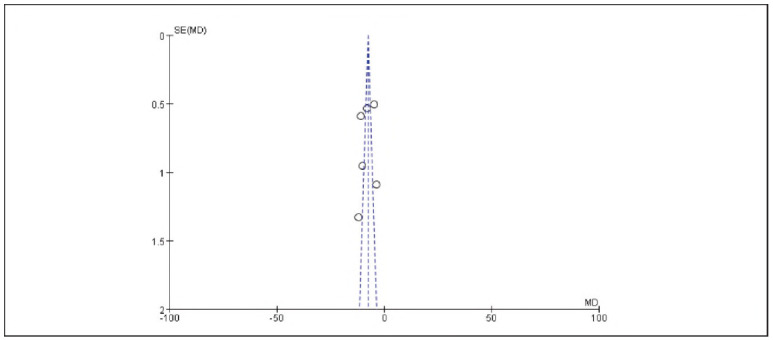
Funnel Plot of Interleukin-6 (IL-6).

These findings suggest that dexmedetomidine reduces inflammatory responses in patients undergoing gastric cancer surgery. See Figure 16, Figure 17, Figure 18-Figure 19.

### Meta-analysis of adverse reaction indicators with dexmedetomidine

Nine studies reporting adverse reactions were included. Heterogeneity analysis showed homogeneity among the studies (I^2^ = 0.0%, P = 0.50). Therefore, a fixed-effects model was applied.

The results demonstrated that the incidence of adverse reactions in the observation group was significantly lower than that in the control group, with a statistically significant difference (RR: 0.37, 95% CI: (0.24, 0.56), P < 0.00001).

**Figure 20:**
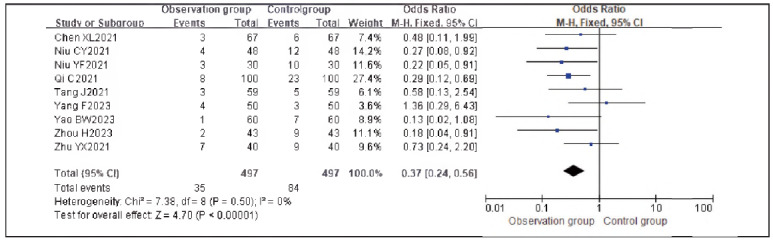
Forest Plot of Adverse Reactions.

**Figure 21:**
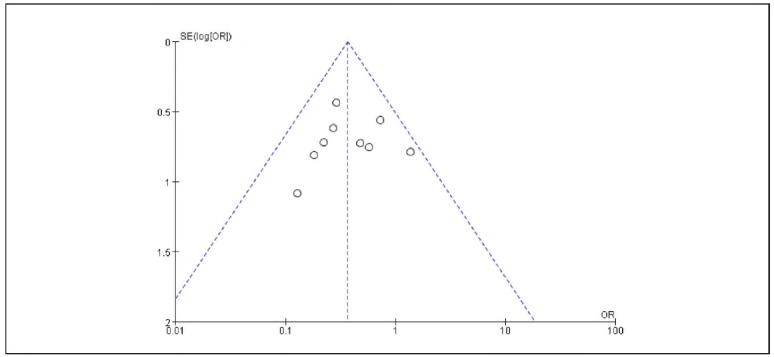
Funnel Plot of Adverse Reactions.

These findings suggest that dexmedetomidine reduces the incidence of adverse reactions in patients undergoing gastric cancer surgery. See Figure 20-Figure 21.

## Discussion

The results of this study showed that the observation group exhibited lower heart rate (HR), mean arterial pressure (MAP), and incidence of adverse reactions compared to the control group. This suggests that dexmedetomidine anesthesia can help maintain hemodynamic stability while effectively reducing adverse reactions. This effect may be attributed to dexmedetomidine's minimal impact on respiratory and circulatory functions, as well as its high lipid solubility, which enables rapid distribution into the bloodstream and easy penetration of the blood-brain barrier. As a result, dexmedetomidine reduces interference with hemodynamics while providing sedative effects. Mechanistically, dexmedetomidine promotes potassium ion influx and contracts peripheral vascular a-receptors, thus counteracting the vasodilatory effects of anesthetics like sevoflurane and enhancing cardiac sympathetic reflexes, which further lowers blood pressure. Through these multiple mechanisms, dexmedetomidine helps maintain relative hemodynamic stability during surgery, thereby enhancing surgical safety and reducing the risk of anesthesia-related adverse reactions [19].

Our data analysis also revealed that the observation group had significantly lower VAS scores compared to the control group, indicating that dexmedetomidine provides effective analgesia for patients undergoing gastric cancer surgery. Dexmedetomidine acts by binding to α-adrenergic receptors in the locus coeruleus, regulating norepinephrine release, maintaining dynamic stability within the central nervous system, and facilitating signal transmission. This mechanism effectively prevents emergence agitation during anesthesia and activates the descending medullospinal noradrenergic pathway, thereby attenuating nociceptive stimuli. As a result, dexmedetomidine lowers postoperative pain thresholds and sensitivity, ultimately enhancing analgesic effects [20].

Surgery stimulates the body and triggers stress responses, activating the hypothalamic-pituitary-adrenal axis and sympathetic-adrenal medullary system. Elevated levels of Cor, epinephrine (E), and ACTH directly reflect the stress state of the body and are highly sensitive indicators. Surgical trauma induces immune suppression and tumor cell immune evasion, leading to inflammatory responses in which IL-6 and TNF-α are involved [21]. Comparative analysis showed that stress and inflammatory markers were significantly lower in the observation group than in the control group, indicating that dexmedetomidine significantly alleviates stress and inflammatory responses. This is mainly because dexmedetomidine modulates pro-apoptotic/anti-apoptotic proteins to inhibit the release of excitatory neurotransmitter glutamate, thereby reducing surgery-induced immune-inflammatory reactions, maintaining dynamic homeostasis, and alleviating immunosuppression, which facilitates postoperative recovery [22]. Moreover, dexmedetomidine acts on the locus coeruleus to regulate GABA neuronal activity, maintaining excitatory-inhibitory balance. Its anti-inflammatory and antioxidant properties reduce the release of inflammatory factors induced by surgical stress. Combined with stress response indicators, dexmedetomidine's antiinflammatory effect suppresses ROS generation, mitigating oxidative stress [23].

In summary, dexmedetomidine anesthesia can reduce the hemodynamic impact on gastric cancer surgery patients, alleviate inflammation and oxidative stress, improve analgesic effects, and does not increase the risk of adverse reactions, warranting wider clinical application. However, this study has limitations, including a relatively small number of included English-language studies, which did not meet the expected target and may have introduced bias and heterogeneity. Furthermore, validating the efficacy and safety of dexmedetomidine in gastric cancer surgery remains a key focus of current research. Therefore, the study design still has room for optimization. Future research should expand literature searches to ensure a sufficient sample size for metaanalysis, thereby improving the reliability and reference value of the results based on higher-quality data.

## Dodatak

### Funding information

This study was supported by the 305 Hospital Independent Scientific Research Fund (No. 25ZZJJLW-009).

### Acknowledgments

This study did not receive any funding in any form.

### Conflict of interest statement

All the authors declare that they have no conflict of interest in this work.

### Contributions

Xuhui Zhang and Wenjun Hu contributed equally to this work.
